# The rhythm of chemotherapy and the felt experience of time: a front-loaded phenomenological retrospective cohort study

**DOI:** 10.1038/s41598-023-35856-4

**Published:** 2023-06-07

**Authors:** Marcin Moskalewicz, Piotr Kordel, Maciej Kokociński, Jadwiga Wiertlewska-Bielarz, Piotr Makowski

**Affiliations:** 1 Phenomenological Psychopathology and Psychotherapy, Psychiatric Clinic, University Hospital Heidelberg, Heidelberg, Germany; 2 Philosophy of Mental Health Unit, Department of Social Sciences and the Humanities, Poznan University of Medical Sciences, Poznan, Poland; 3 Institute of Philosophy, Marie Curie-Sklodowska University, Lublin, Poland; 4 IDEAS NCBR, Warsaw, Poland; 5 Faculty of Sociology, Adam Mickiewicz University in Poznan, Poznan, Poland; 6 Faculty of Management, University of Warsaw, Warsaw, Poland; 7 Queen’s University, Belfast, UK

**Keywords:** Psychology, Cancer therapy, Quality of life

## Abstract

It is well-known that chemotherapy brings about various adverse physical effects such as fatigue, nausea, or vomiting, and that it lowers mental well-being. It is less known that it desynchronizes patients with social environment. This study explores the temporal aspects and challenges of chemotherapy. Three groups equal in size and distinguished according to weekly, biweekly, and triweekly treatment schemes, each independently representative in terms of sex and age of the cancer population (total N = 440) were compared. The study found that chemotherapy sessions, regardless of their frequency, patients’ age, and the overall length of treatment, have a very large effect on changing the felt pace of time from flying to dragging (Cohen’s d = 1.6655). Most patients pay more attention to the passing of time than before treatment (59.3%), which has to do with the disease (77.4%). They also experience the loss of control over time, which they subsequently attempt to regain. The patients’ actual activities before and after chemotherapy, however, are mostly the same. All these aspects create a unique 'chemo-rhythm', in which the significance of the type of cancer and demographic variables is negligible, and the mere rhythmic nature of treatment plays a central role. In conclusion, patients find the ‘chemo-rhythm’ stressful, unpleasant and difficult to control. It is vital to prepare them for it and help to reduce its adverse effects.

## Introduction

### Time and illness

We used to believe that the saying “time heals all wounds” is true. However, there are clear cases in which the beneficial effects of time are uncertain or even non-existent. The most obvious is emergency medicine, where time almost always works against us. Another example is chronic diseases defined by the prolonged nature of symptoms and treatment. Patients suffering from enduring ailments, especially severe ones like cancer, must deal with painful, unpleasant, and stressful situations for a long time. Although it is evident that the sheer prolonged duration of illness is a challenge for patients, it has significant consequences for temporal experience. In such chronic conditions, time is often disrupted and becomes an additional factor of illness^1–4^.

This study deals with the impact of chemotherapy on cancer patients' felt sense of time. Unlike in most theories of time perception, the felt sense is inextricable from personal identity in illness^5^. One of the most conspicuous features of such a felt sense of time is its frequent acceleration and deceleration regarding the environment. When lived in synchrony with the world, time is implicit—it is not directly perceived but constitutes a tacit background of conscious experience. On the other hand, phenomenological psychopathology posits that time becomes explicit or directly perceived when one notices asynchrony between oneself and the world. This manifests as either the sense of time flying or dragging ("having" too little or too much time, respectively)^6^. One may draw a spectrum of moods corresponding to those experiences stretching from hectic pace and agitation, the pressure of time and impatience, to boredom, fatigue, guilt, and melancholia^7^. Positive emotions associated with a pleasant event in the future imply the expansion of time, while negative emotions such as frustration give the experience of time contraction^8^. When neither acceleration nor deceleration exists, time 'disappears' as an object of experience. It becomes implicit, to use a phenomenological term, and one experiences only its tacit flow.

Despite its abstract character, these issues are of high practical relevance. The association between emotional well-being and temporal perspective on the future has been studied for decades. The felt sense of time may be a significant source of stress and discomfort for oncological patients^9,10^. There is strong evidence that an optimistic outlook provides a variety of emotional, social, and health benefits for people with cancer^11–13^. For example, in breast cancer patients, positive expectancies are an important cognitive mechanism by which they continue to pursue valued life goals^12^. Conversely, past ruminations are associated with psychological distress^11^. An unusual high focus on the future among cancer patients might be a coping mechanism that helps to maintain motivation to fight the disease^12^.

### Qualities and quantities of lived time

The experience of time flying or dragging concerns a dimension of time that has to do with its felt flow in the present and not with a more structural focus on its different dimensions as indicated above^13^. Furthermore, this experience must not mirror the cognitive perception of time in objectifying terms as measured against the clock in experimental tasks, which are also applied to measure time perception in cancer^14–16^. Events whose temporal length one correctly estimates may drag or pass quickly nevertheless, and events whose temporal length one over- or underestimates may not be felt as decelerated or accelerated^17,18^. The phenomenological distinction mentioned above between implicit and explicit time is akin to the one between retrospective and prospective paradigms used in experimental tasks on time perception. However, while the explicitness of time is a condition for executing prospective tasks, the explicitness itself cannot be measured in terms of the 'amounts' of time passed^17^.

Classic phenomenology has studied lived time qualitatively, that it its felt-sense and its structures, regardless of objectified time 'amounts'^19^. It was not interested in quantification, which was one of its limitations from the perspectives of the goal of this study. Our approach posits that the felt qualities of lived time, which are phenomenological in nature, may nevertheless be *quantified* and analysed as quasi-amounts via a Likert scale. What is being measured is no longer time understood as a homogenous series of events on the clock, but its *felt experience*. Therefore, this study aims at quantifying some phenomenologically pre-conceived aspects of lived time. To the extent that the study combines a focus on lived experience characteristic of many standard phenomenological approaches with hypotheses testing characteristic of natural science, its method may be understood as quantitative-phenomenological or front-loaded phenomenological—the latter term referring to Gallagher’s idea to front load insights developed in phenomenological analysis into the design of empirical work^20^. Analogical mixed approaches have already appeared in the literature^21,22^ and was also tested by us previously^23^.

### Aims of the study

This research aims to quantitatively validate and explore qualitative phenomena uncovered in our previous study of lived temporal experience of 9 ovarian cancer patients during chemotherapy—now on a sample of 440 service users suffering from various types of cancer. The earlier qualitative study combined Giorgi's method of descriptive phenomenological psychology and consensual qualitative research. Its key finding concerned the so-called temporal paradox of chemotherapy^24,25^. In accordance with the method employed, this paradox was defined as an essential psychological structure (what phenomenological philosophy refers to as the eidetic background) that organized the respondents' experience from behind or implicitly. This structure was common to the group despite different individual manifestations. In other words, it left traces on how patients experienced their world in time, without being itself seen by them as such.

### Previous qualitative study results

The temporal paradox of chemotherapy was that ovarian cancer patients lived from chemo to chemo, which in that case meant in three-weeks rounds, with hospital visits appearing as their primary *Zeitgeber*. On the one hand, the three-weekly rhythm organized their temporal experience, but on the other, it rendered it insecure and disturbed. As one patient put it:

I live from appointment to appointment^24^.

In between the appointments, the patients reported experiencing deceleration and acceleration, the former after the chemo and the latter before the upcoming chemo. It was a recurring process through which felt time first "stretched" and then "shrunk". As is well-known, and as one of the interviewed patients succinctly put it:

When one suffers, it drags. And when one is better it flies^25^.

Here, however, the process was found to be rhythmic in nature and ruled by the administration of chemo and the concurrent significance of the hospital visits. In addition, the patients felt confused by the changing pace of time and felt the need to regain control over it. After chemo, they struggled to find activities that would accelerate dragging time, and before chemo they fantasized about decelerating flying time. As another patient put it:

I am always looking at my watch, when will the day pass? To feel better. (…) And then after this Friday, this first week, I am like, oh God, let it fly slowly, so that I don't have to go. (…). It is divided by half [laughs]. I want it to pass quickly and feel better and then (…) I am saying, please don't fly^24^.

Therefore, the very rhythm of chemotherapy was crucial for the apparent deformations of the patients' felt experience of time. Would this structure operate in other types of cancer and with different treatment frequencies was the primary question of interest.

### Hypotheses

Based on these initial qualitative phenomenological findings, we hypothesized that:

1. After chemotherapy, patients experience time dragging and the concomitant need to accelerate it, while before chemotherapy, they experience time flying and the concomitant need to slow it down.
2. The experience of acceleration and deceleration presupposes the explicitness of time, which means that time ceases to be a tacit background of conscious experience and becomes an object of perception. We, therefore, hypothesized that time must become explicit for the patients around chemotherapy sessions.
3. Finally, we speculated that patients lose control over time right after chemo and then slowly, progressively regain this control up to their next chemo.

We aimed to check whether these three phenomena can be observed in a larger sample of cancer patients and how frequent they are. We also wanted to find out if they correlate with the frequency of chemotherapy sessions, time elapsed since the diagnosis and the beginning of treatment, and the socio-demographic characteristics of the service users.

### Agency and time management

Expanding on these hypothesis, we have added a more exploratory part to the study that elaborates on the level of agency in time management understood as an implementation of self-regulation processes in the temporal domain. Acknowledging that time management is affected by increased stress^26^, we assumed that time acceleration and deceleration should weaken and destabilize those self-regulation processes. The overall assumption stemming from our qualitative study was that the rhythms of chemotherapy are the key determining factor responsible for the patients' temporal ups and downs and the concomitant distress, and not any social, economic, or demographic factors.

While the impact of rhythms on behaviour has been widely studied in many research fields under various headings, e.g. habits in psychology^27,28^, habituses and practices in sociology and social theory^29–31^, rhythms, routines and organisational practices in management research^32,33^, surprisingly little has been done in psycho-oncology. The emerging research is limited to habits^34^, and it does not discuss the question of agency and time management, which appears to be crucial as far as the rhythmic nature of chemotherapy is concerned. Also, the rhythms of chemo and their impact on time management appear to be different from the influence of patterns and repetitiveness of habits on behaviour. Lessons from normal routine functioning in organizational settings suggest that rhythms—understood as characteristic successions of the rate of performed activities have clear consequences for time management. They allow agents to achieve *temporal autonomy*, i.e., to temporally uncouple from the unfolding situation to maintain adaptability to autonomously selected events^32^. In the rhythms of chemotherapy, the temporal scenario appears to be much more complicated. The only two studies concerning this subject were conducted by the authors of this paper (MM, PK, and JWB). One attempted to measure the impact of rhythms on cancer patients' temporal perspectives and found that the increased frequencies lower the level of hedonism^12^. The other focused on the frequencies of chemotherapy found that they are inversely related to patients' orientation in time and their awareness of mortality, both being the highest for those in the triweekly treatment^23^.

## Methods

### Quota-purposive sampling

Since the Polish cancer patients population size is approximately 1.17 million service users, the study aimed at a sample larger than 384^35^ General inclusion criteria: adult service users had to be diagnosed with any type of cancer for at least a month and undergo out-patient chemotherapy (be at least after two cycles). We excluded patients in hospice, home or in-patient treatment, but not the patients receiving palliative care. Curative and palliative patients were thus brought together in the sample on the basis of their functioning and not being confined to their beds, and their responses were not coded separately.

The respondents had the following cancers: colon 38,4%, breast 29,1%, lung 7%, pancreatic 5,5%, stomach 5%, kidney 2,3%, prostate 1,8%, liver 1,6%, ovarian 1,4%, and other 8%. The mean age of respondents was 62.37 years (Me 66.0, SD 11.70), the mean time since diagnosis was 23.01 months (Me 12.00, SD 32.43), and the mean time since the beginning of chemotherapy was 14.57 months (Me 8.00, SD 21.32).

The key to purposive sampling was distinguishing three groups according to weekly, biweekly, and triweekly or less frequent treatment schemes, where different frequencies were treated as different exposures factors. Since data on the number of service users’ chemotherapy frequency was unavailable, the number of respondents recruited to each of the three subsamples was similar (N1 = 150, N2 = 146, N3 = 144, Total N = 440; see Table 1). Each of these subsamples was independently representative in terms of sex (m:f ratio of 1:1) and age (m > 65 = 61%, f > 65 = 53%) of the Polish cancer population (according to The Polish National Cancer Registry 2017 statistical report). These quotas represent the global cancer population according to Global Cancer Observatory data (2020) for sex (m:f ratio of 1:1) and age for females, while for men the sample deviates by 4% (for the global population: m > 65 = 57%). The threshold of 65 years was chosen for society-specific reasons; namely, the age of retirement in Poland that likely impacts the felt experience of time. It is also the peak of cancer morbidity distribution in this population.

**Table 1:** Quota sampling regarding age, sex, and treatment frequency (N = 440).

		Age > 65		Age < 65		Total
		Female	Male	Female	Male	
Chemo-rhythm						
Weekly	N	40	46	34	30	150
	%	9.1%	10.5%	7.7%	6.8%	34.1%
Biweekly	N	34	44	34	28	146
	%	9.1%	10.0%	7.7%	6.4%	33.2%
Triweekly or less frequent	N	38	28	34	28	144
	%	8.6%	10.0%	7.7%	6.4%	32.7%
Total	N	118	134	102	86	440
	%	26.82%	30.45%	23.18%	19.55%	100%
Mean age (years)		70.1	70.4	51	52.8	

### Research survey

Patients filled in three questionnaires. The first was demographic and contained questions regarding sex, age, place of residence, education, marital and professional status, disposable income, religion, type of cancer, time elapsed since diagnosis and the beginning of treatment. The second was a 10-item research survey with questions concerning three different aspects of the felt sense of time (deceleration/acceleration, explicitness, and losing and regaining control), which was designed based on previous qualitative study results concerning ovarian cancer patients' lived experiences.

The respondents were asked if they experience the feeling of time flying (up to three days before chemo sessions) and dragging (up to three days after a chemo session) and whether they wish to counteract those feelings (these four questions were formatted in a 7-point Likert scale). The patients were also asked about the explicitness of time, that is, whether they are more aware of the passing of time (a single 7-point Likert scale question) and in which circumstances this feeling appears (a nominal scale question). Finally, they were asked about their use of time while in treatment, whether they feel that time is completely lost after chemo sessions, and whether they are able to make better use of it if more time has passed since their last chemo session (two questions formatted in 7-point Likert scale). All the above questions were aimed at testing hypotheses stemming from the previous qualitative results.

In addition, the explorative part of the study on agency and time management was based a 25-items list of possible activities before and after chemo that the respondents could chose from. The classification of activities was based on our previous interviews with ovarian cancer patients and the knowledge of the decline of physical activity during cancer treatment^36,37^. The list included activities requiring physical exertion (in the form of daily chores, such as household duties, or extra activities such as sports), activities involving the presence of others (the interviewees emphasized the importance of loved ones, and we supplemented it with activities with people with less emotional ties such as social gatherings), and activities involving cognitive faculties. Among the latter, we distinguished those that are indispensable and useful (dealing with bills), not necessarily useful (daydreaming), and merely recreational (puzzles). The complete list contained the following options: planning the future, filling in forms, arranging documents, dealing with bills, studying, religious practices, daydreaming, reading, conversations with family/friends, games, puzzles, watching tv, social gatherings, creative work, DIY, shopping, household duties, renovations, taking care of someone, taking care of one's appearance, recreational sports, going for a walk, dancing, sexual activity, gardening, trips.

### Survey validation

The 10-item survey and the the 25-items list were first face-validated by researchers (MM, PK, and JWB) and second by experts by experience (five patients during chemotherapy, who independently assessed the internal validity of questions). Subsequently, some of the questions were reworded for comprehensibility of meanings related to the lived experience of time. A further pilot study of 20 service users with cancer contained some control questions to assess their understandability. As a result of the feedback received, the final version of the survey (as described above) was developed. The questions on acceleration and deceleration had a good reliability (Cronbach's alpha = 0.778) and the questions on time control a sufficient reliability (Cronbach's alpha = 0.603).

### Data collection

Data was collected at the outpatient care units in Greater Poland Cancer Center and the Chemotherapy Ward of the Transfiguration University Clinical Hospital in Poznan (Poland). Even though the frequencies of treatment varied, all patients filled out the survey at the same stage within their treatment cycle, that is during or shortly after a chemotherapy session. As mentioned above, patients based their answers on at least two cycles of chemotherapy, but since their median time in treatment was eight months, it was at least several cycles for most. The Greater Poland Cancer Centre Bioethics Committee and Poznan University of Medical Sciences Bioethics Committee approved the project. The study was conducted in accordance with the principles of the Declaration of Helsinki, and all patients provided written informed consent prior to enrolment.

### Data analysis

Even though data on temporal experience were collected cross-sectionally, the study can be considered a variation of a retrospective cohort study because time was also treated as an exposure factor. More precisely, how patients experience time in chemotherapy (both their felt sense of time flying or dragging, their explicit awareness of its passing, and their losing and regaining control over it) was hypothesised to be an outcome of either the amount of clock time passed since birth (age defined in years) or the beginning of chemo (the length of treatment defined in months) or the frequency of treatment (defined in weeks as either weekly, biweekly, or triweekly). In this way, linear and circular time could be compared as different exposure factors (independent variables), and their significance for several dimensions of the felt experience of time (dependent variables) could be assessed.

### Ethics approval

Bioethics committees at target institutions approved the research: Greater Poland Oncological Center – decision from 11.09.2020; Transfiguration University Clinical Hospital in Poznan – approval number LBK 117/2020.

## Results

### Deceleration and acceleration

The study found that patients experience time dragging and the concomitant need to accelerate it after chemotherapy, and time flying and the concomitant need to slow it down before chemotherapy. It thus confirmed and expanded the previous qualitative finding concerning the temporal paradox of chemotherapy.

48.2% of the 440 sample confirmed (answered rather yes, yes, or strongly yes) that their felt time drags after treatment and 70% that their felt time flies before treatment. In order to precisely assess the effect the chemotherapy on patients’ felt deceleration and acceleration, we recoded the two 7-point Likert scales answers into a single scale of the felt pace of time, ranging from − 4 to + 4, where 4 represented a response “strongly yes” to the question *Before the chemotherapy session *(*1–3 days*), *I have a feeling of time flying,* and − 4 represented a response ‘strongly yes’ to the question *After a chemotherapy session *(*1–3 days*), *I have a feeling of time dragging.* All the negative answers (strongly no, no, and rather no) indicating the lack of felt acceleration or deceleration were recoded as 0. In this way, the analysis could capture not only the alternations of patients’ felt time but also asses the change of direction of the alternation affected by chemotherapy.

**Figure 1:**
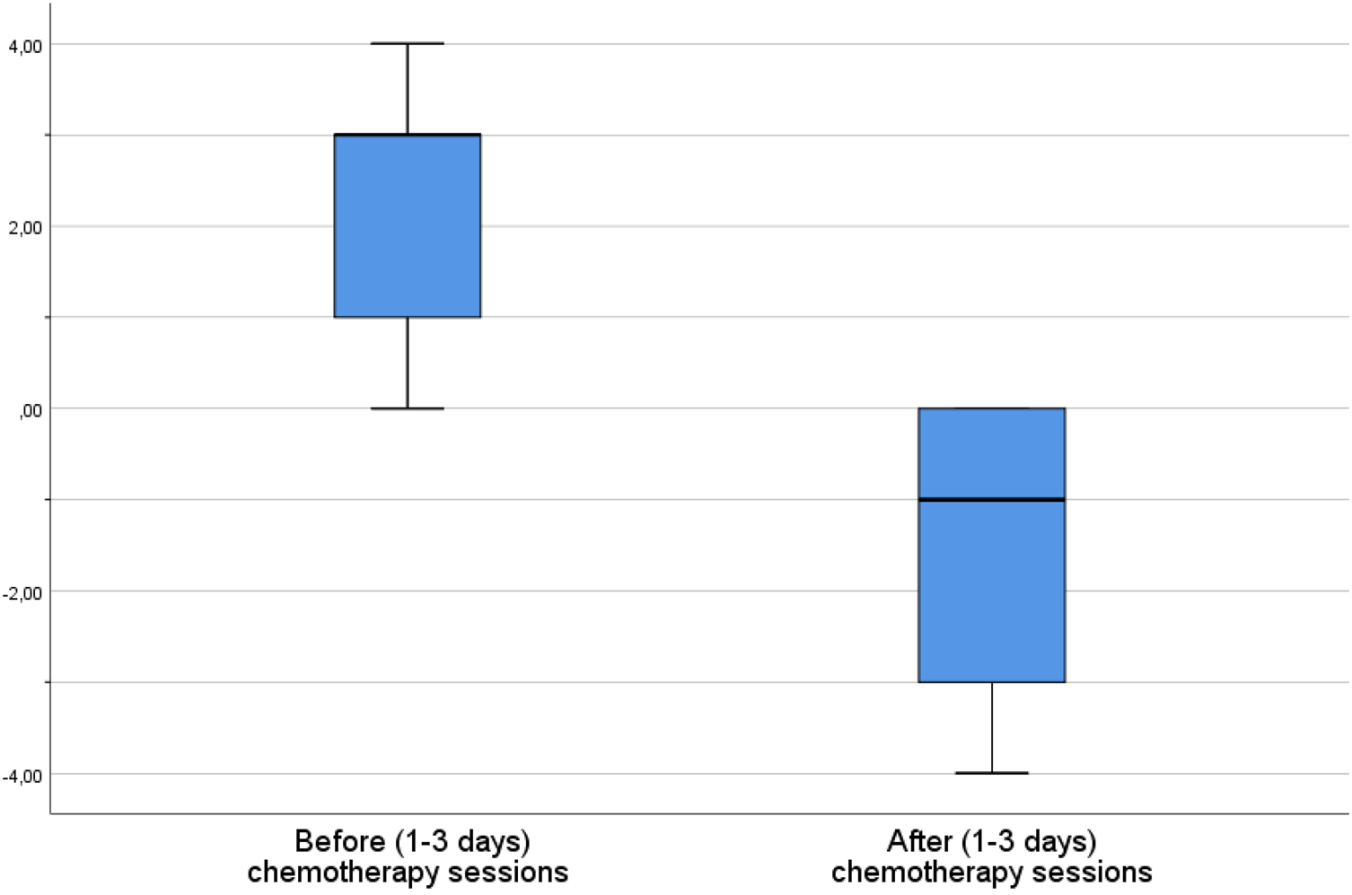
Felt time alternations before and after chemotherapy sessions, N = 440. − 4 = a strong feeling of time dragging, 4 = a strong feeling of time flying. *t(439) = 43.543; *p* < 0.001, Cohen’s d = 1.6655.

Figure 1 shows that the feeling of time flying before chemo is stronger than the feeling of time dragging after and that the difference is statistically significant. But what is more important, the chemotherapy sessions have a very large effect on the patients’ experience of the felt pace of time (Cohen’s d = 1.6655). The feeling of time flying is stronger among female patients (5.341 [SD 1.567] vs. 4.955 [SD 1.568], Mann–Whiney's U = 20,372, η^2^ = 0.02, *p* = 0.003), which can be attributed to the fact that in Poland women tend to work more in the households, pay more attention to maintaining social relations, and care more about their appearance. Hence, they might want to do more before the upcoming chemo session. Nevertheless, in this case the effect size is very low.

**Figure 2:**
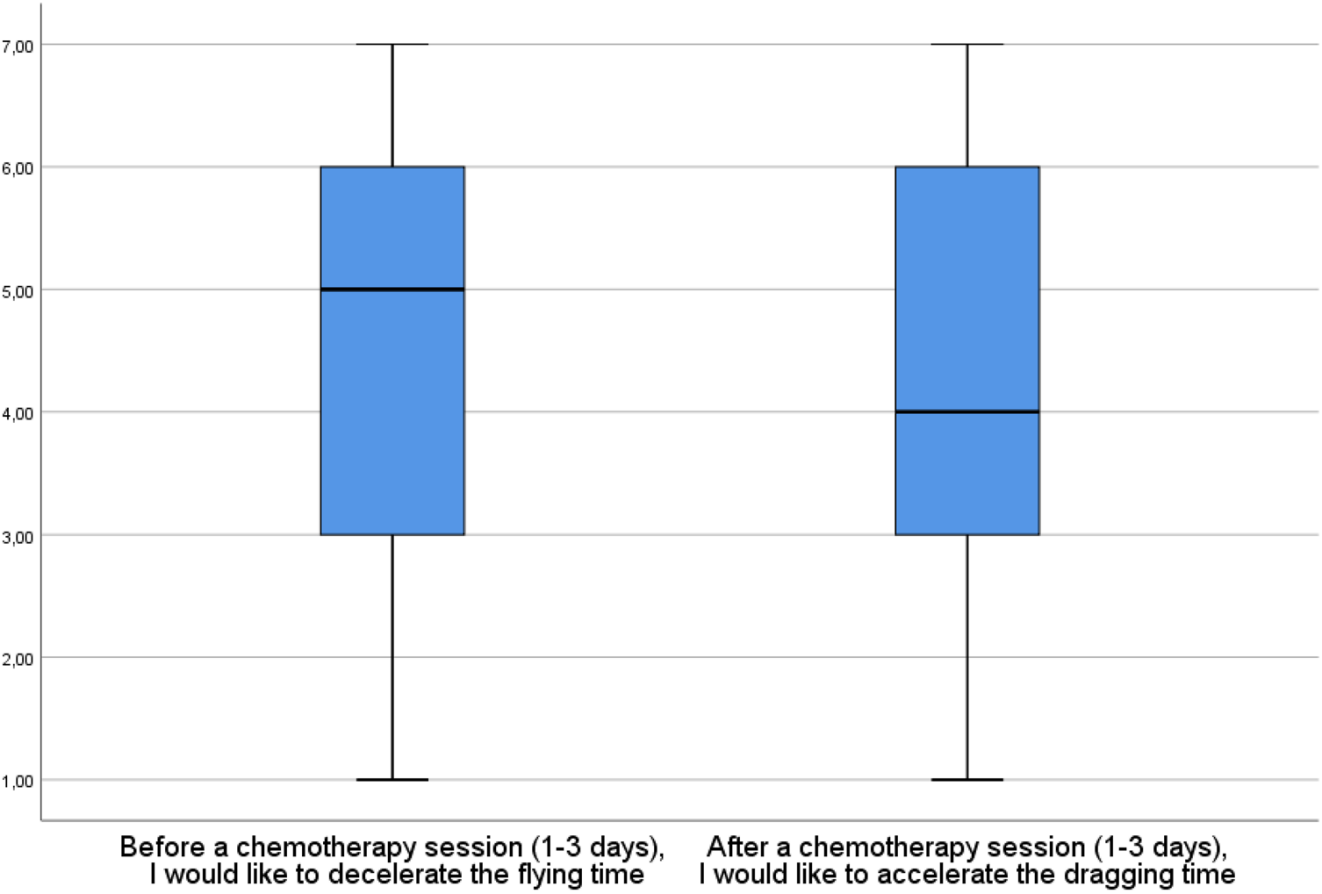
The need to counteract felt time alternations before and after chemotherapy (N = 440). 7 = I strongly agree; 1 = I strongly disagree; t(439) = 2.583; *p* = 0.01, Cohen’s d = 0.12.

When it comes to the need to counteract the feelings of time dragging and flying, the need to decelerate time before chemotherapy sessions is stronger than the need to accelerate time after chemo, although the effect size is again very low (see Fig. 2).

Most importantly, the frequency of treatment, weekly, biweekly or triweekly, does not differentiate the felt sense of time flying or dragging and the need to counteract these experiences (*p* > 0.05). Furthermore, neither of these phenomena correlates with linear time since treatment (*p* > 0.05), the patients' age (r_s_ = 0.108, *p* = 0.023—a correlation so weak that can be treated as non-existent) nor with the type of cancer (*p* > 0.05). In other words, and crucially, it is only the rhythmic nature of treatment, and not its frequency nor duration, that affects the patients' felt acceleration or deceleration of time.

**Figure 3:**
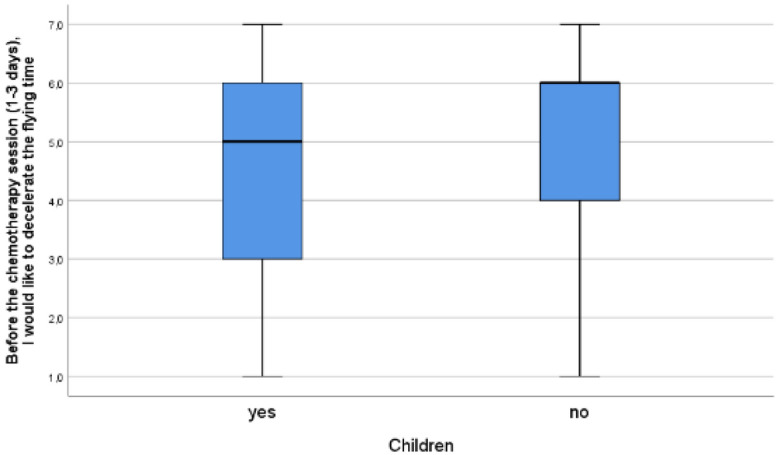
Having children and the need to decelerate time before chemo. 7 = I strongly agree; 1 = I strongly disagree; Mann–Whitney’s U = 12,752,5, η^2^ = 0.019, *p* = 0.003.

**Figure 4:**
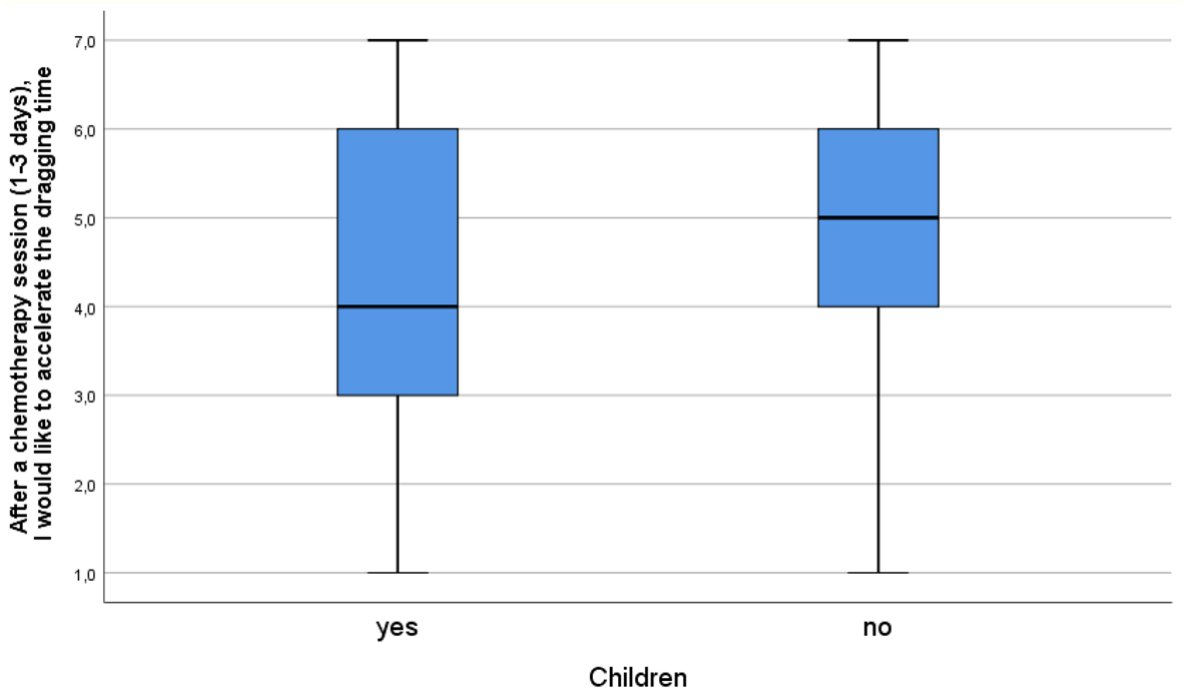
Having children and the need to accelerate time after chemo. 7 = I strongly agree; 1 = I strongly disagree; Mann–Whitney’s U = 12,381, η^2^ = 0.025, *p* = 0.001.

Furthermore, there is a moderate correlation between the feeling of time dragging and the need to accelerate it and between the feeling of time flying and the need to decelerate it (accordingly r_s_ = 0.532, *p* < 0.01, and r_s_ = 0.499, *p* < 0.001). This indicates that those feelings are problematic and unpleasant, which is not self-evident, thus confirming our hypothesis from the qualitative study. Somewhat counterintuitively, time flying appears to be more problematic than time dragging. It is because the feeling of time flying before chemo is stronger than the feeling of time dragging after chemo (see Fig. 1), and the need to decelerate time before chemotherapy sessions is stronger than the need to accelerate time after chemo (see Fig. 2) while the correlations between both types of felt time disruptions and the desire to counteract them are equally strong (r_s_ = 0.532 and r_s_ = 0.499). It is also worth adding that these experiences do not correlate with linear time variables (age and time elapsed since diagnosis and beginning of treatment, *p* > 0.05). Childless patients experience this more intensively but the effect size is very low (see Figs. 3, 4).

### Explicitness of time

Time becomes explicit when it ceases to be a tacit background of conscious experience and service users notice its passing. It occupies their conscious attention. The study confirmed that time becomes more explicit in treatment and around chemo sessions. 59.3% of respondents pay more attention to its passing than before the beginning of treatment, against 22.1% who don't (mean 4.814, SD 1.666). Patients notice time passing most often during a chemo session and/or shortly after (26.8%), when it decelerates, and just before the next chemo session (27%), that is when it accelerates. 23.6% of respondents indicated that time occupies their attention in other circumstances related to the disease.

When it comes to the socio-demographic features that correlate with the explicitness of passing time, Mann–Whitney's U test results show significant differences (*p* < 0.05) only between men and women (mean, [SD] 4.7 [1.55] vs. 4.93 [1.77]) and patients having children and childless (4.75 [1.66] vs. 5.12 [1.65]). However, the explicitness of time does not correlate with the crucial variable of linear time [counted either from the beginning of treatment or as age (*p* > 0.05)], and it does not correlate with different chemo-rhythms (*p* > 0.05). Perhaps such increased awareness of the passing of time has mostly to do with entering the treatment phase, regardless of how long it lasted, how intensive it is, and how old the patient is.

### Losing and regaining control over time

**Figure 5:**
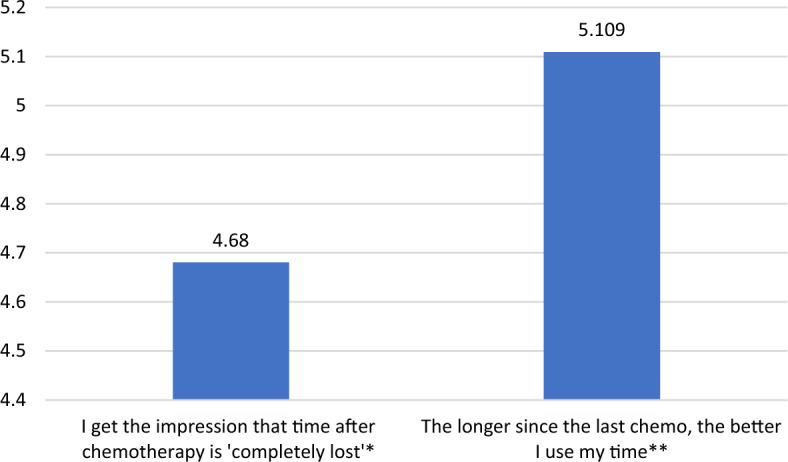
Losing/regaining control over time after/before chemotherapy (means, N = 440). 7 = I strongly agree; 1 = I strongly disagree ;* SD = 1.785, **SD = 1.5647, t(878) = 3.849; *p* < 0.0001, Cohen’s d = 0.26.

Following the qualitative study results, we speculated that patients lose control over time right after chemo and then slowly, progressively, regain it—up to their next chemo. Indeed, 58% of respondents find time after chemo "completely lost". They agree with the statement that the longer since the last chemo, the better they use time, more (5.109) than with the statement that after chemotherapy their time is completely lost (4.68)—see Fig. 5.

Crucially, both feelings regarding time, that is 'losing it' after treatment and then progressively regaining, do not correlate with the actual linear time passed since the beginning of treatment nor the patients' age (*p* > 0.05). However, unlike with the felt acceleration/deceleration and the explicitness of time, chemo-rhythm is statistically significant for the better use of time, which is the highest for the triweekly group (see Table 2). Perhaps, since more time between sessions is available to patients undergoing triweekly treatment, they are more likely to recover from its adverse effects. This suggests that patients whose sessions are less frequent are able to deal with time dragging and time flying within the normal limits of temporal autonomy.

**Table 2:** Chemo-rhythm and regaining control of time.

	Chemo-rhythm	N	Mean	SD	Median
(A)					
The longer since the last chemo, the better I use of time	Weekly	150	4.860	1.5887	5
	Biweekly	146	5.130	1.5325	6
	Triweekly	144	5.347	1.5434	6
	Total	440	5.109	1.5647	5

Weekly vs. biweekly	Weekly vs. triweekly	Biweekly vs. triweekly			
(B)					
Dunn's post-hoc test results					
*p* > 0.05	***p* = 0.014**	*p* > 0.05			

(A) 7 = I strongly agree; 1 = I strongly disagree; Kruskal–Wallis H test χ2 = 8.039, *df* = 2, *p* < 0.05.

(B) Dunn Test = − 40.9.

Significant values are in bold.

**Figure 6:**
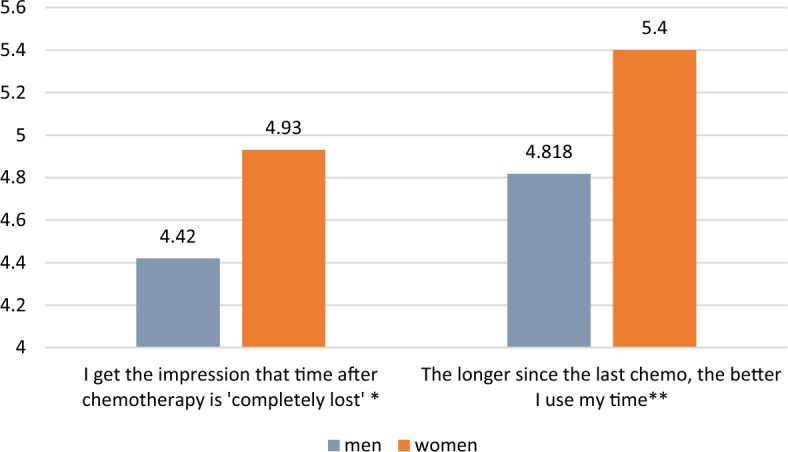
Sex and losing/regaining control over time after/before chemotherapy (means, N = 440). 7 = I strongly agree; 1 = I strongly disagree ;*Mann–Whitney’s U = 20,048, η^2^ = 0.022, *p* = 0.002; **Mann–Whitney’s U = 19,069, η^2^ = 0.035, *p* < 0.0001.

**Figure 7:**
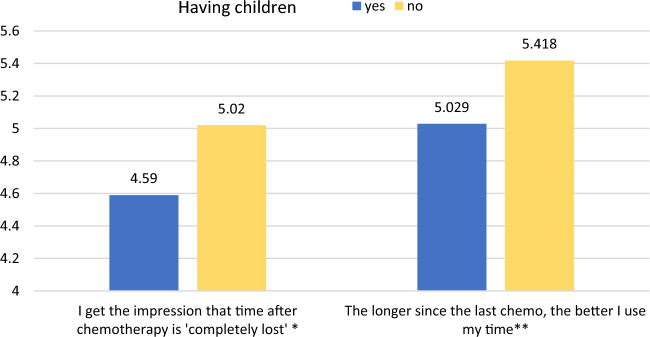
Having children and losing/regaining control over time after/before chemotherapy (means, N = 440). 7 = I strongly agree; 1 = I strongly disagree ; *Mann–Whitney’s U = 13,390, η^2^ = 0.012, *p* = 0.019; ** Mann–Whitney’s U = 13,633.5, η^2^ = 0.01, *p* = 0.033.

Furthermore, women and childless patients experience losing time after chemotherapy and regaining control over it more intensively than men and patients with children, but size effects are again very low (see Figs. 6, 7).

### Agency and time management

**Figure 8:**
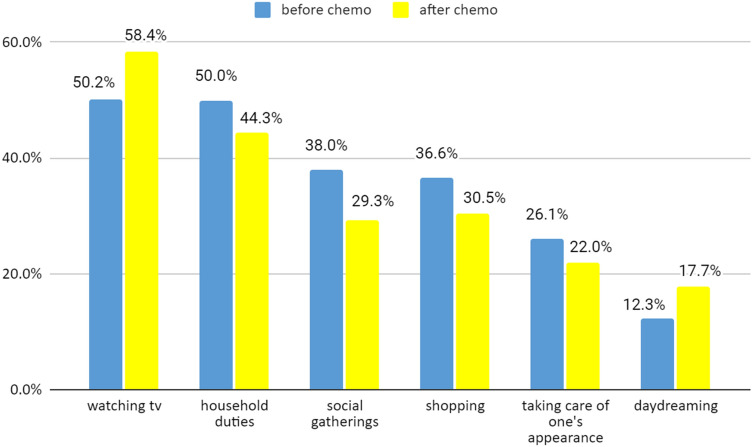
Patients' activities differing before and after chemo (McNemar's Change Test *p* < 0.05, N = 440).

The exploratory part of the study showed that despite the felt acceleration and deceleration as well as losing and gaining control over time, the respondents' actual activities before and after chemotherapy are mostly the same. The McNemar's Change Test results indicate that the incidence of activities differed significantly in only 6 out of 25 listed actions. Patients practiced physically less engaging activities such as watching tv and daydreaming more often after chemo than before (58.4% vs. 50.2% and 17.7% vs. 12.3.%, respectively). On the other hand, they were taking care of their appearance or their household, shopping, and partaking in social gatherings more often before chemo than after (26.1% vs. 22.0%, 50% vs. 44.3%, 36.6% vs. 30.5%, 38% vs. 29.3% respectively; see Fig. 8). Watching TV and household duties were also among the most frequent activities overall; more than 40% of the patients were also engaged in conversations with family and friends and strolling. For all other activities, that is, planning the future, filling in forms, arranging documents, dealing with bills, studying, religious practices, reading, conversations with family/friends, games, puzzles, creative work, DIY, renovations, taking care of someone, recreational sports, going for a walk, dancing, sexual activity, gardening, trips, there were no incidence differences between before and after treatment (see Table 3).

**Table 3:** The incidence of patients' activities before and after chemo (N = 440).

	Before chemo (%)	After chemo (%)
Watching tv	50.2	58.4
Conversations with family/friends	45.7	48.6
Going for a walk	48.6	48
Household duties	50	44.3
Reading	30.2	31.4
Shopping	36.6	30.5
Gardening	30	30
Social gatherings	38	29.3
Games, puzzles	26.8	26.6
Arranging documents	24.1	22.7
Planning the future	20.5	22.3
Religious practices	25.5	22.3
Taking care of one's appearance	26.1	22
Taking care of someone	19.1	21.6
Daydreaming	12.3	17.7
DIY	13.9	14.1
Dealing with bills	15.9	13.9
Trips	13.9	13.9
Creative work	10.7	12
Recreational sports	13.2	11.1
Filling in forms	12.3	10.5
Studying	8.4	9.3
Renovations	6.1	6.4
Sexual activity	8.2	6.4
Dancing	3	2.5

Contrary to our initial hypothesis, this might suggest that patients' time management skills were sufficient to deal with their temporal predicament. One may speculate that engaging in physically less demanding activities is one of the methods to retain normal temporal autonomy.

Regarding treatment rhythm, when it comes to the actual activities reported, there are only few and likely negligible differences between the weekly and the triweekly treatment groups. After chemo, the triweekly group more often watches tv (62.7% vs. 50%; Mann–Whitney's U = 9300.00, *p* = 0.029) and takes part in trips (18.3% vs. 9.3%, Mann–Whitney's U = 9694.00, *p* = 0.026). Before chemo, the triweekly group is more often engaged in daydreaming (18.3% vs. 9.3%, Mann–Whitney's U = 9717.00, *p* = 0.029) and playing games (35% vs. 21.5%, Mann–Whitney's U = 9216.00, *p* = 0.01). A very low contingency coefficients (around 0.1 for all the abovementioned activities) also suggests that these data sets are largely independent. As was the case with both the felt acceleration and deceleration and the explicitness of time, it is the rhythmic nature of treatment, and not its frequency, that proves more relevant.

## Discussion

This study aimed to explore the impact of chemotherapy on cancer patients' felt sense of time, in particular the experience of acceleration and deceleration, time explicitness around chemo sessions, and the loss and regaining of control over time in between of the sessions. The larger premise of the research was the hypothetical impact of different treatment rhythms on cancer patients' lived experience of time.

The study found that exposure to different chemo rhythms (weekly, biweekly, triweekly) affects neither the felt sense of time dragging or flying and the need to accelerate or decelerate it, nor the explicitness of time. Therefore, the felt sense of the changing pace of time and its explicitness presumably stem from the very rhythm regardless of its frequency. Moreover, and surprisingly, the type of cancer has no significance for any of the variables of interest, which also confirmed the leading presupposition. When it comes to all demographic variables, even in the case of those few differentiating the results, such as sex for time flying, its explicitness, and losing/regaining control, and having children for the need to accelerate/decelerate time, its explicitness, and losing/regaining control, the effect size is very low and thus their relevance is negligible. It follows that the chemotherapy somewhat “equalizes” the patients and distorts their lived time regardless of their social status and background.

**Figure 9:**
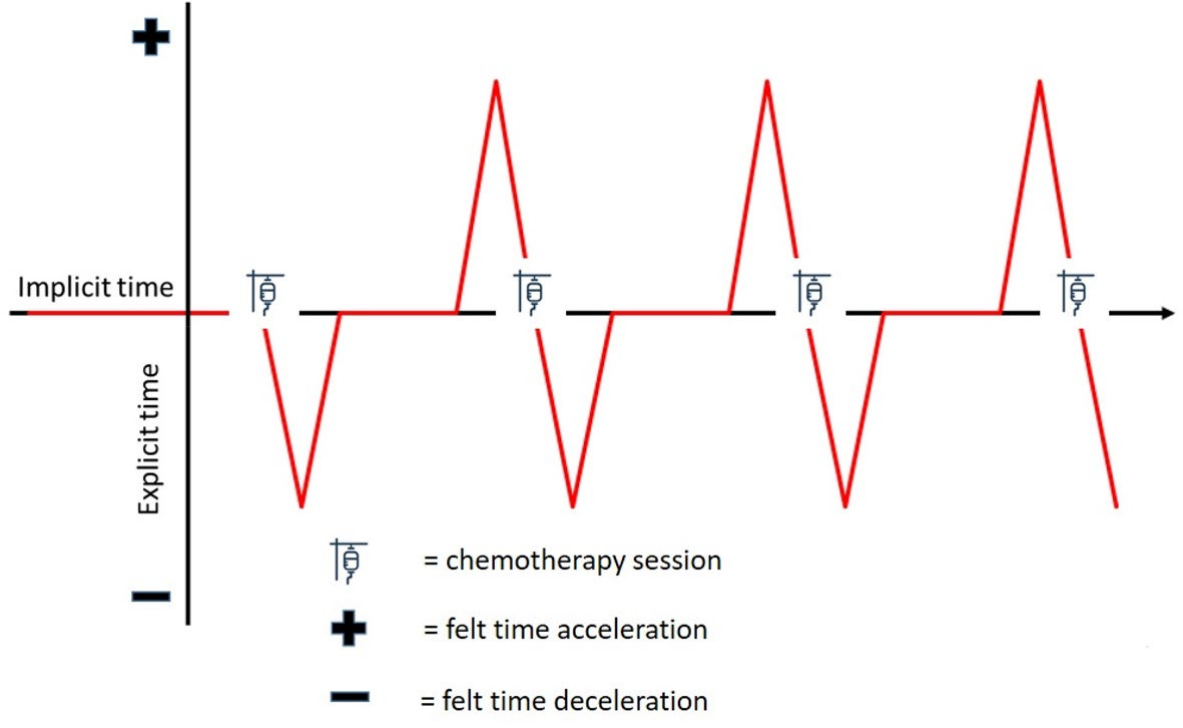
Felt time dragging ( +) after chemo and felt time flying (-) before chemo.

Figure 9 schematically presents the oscillation of acceleration and deceleration between the chemo sessions regardless of their frequency. Since patients were approached at a specific point in time only once, the figure extrapolates cross-sectional data onto the timeline. Both felt acceleration and deceleration presuppose that time becomes explicit for the patients. The figure speculatively presents the time between chemo sessions as implicit for clarity only.

The patients' simultaneous need to accelerate or decelerate the felt flow of time indicates that the ups and downs of the felt temporal flow between chemo cycles are unpleasant for them, and that they likely struggle to find balance. The themes from the previous qualitative study concerning the paradox of time and losing and gaining control over time thus find a quantitative confirmation beyond ovarian cancer and its triweekly treatment plan.

Furthermore, patients' increased thinking about the passing of time reveals another aspect of psychological distress. Patients think more often about time after than before treatment (as previous research also showed^23,24^), and this increased awareness takes place around chemo sessions. However, it is not significantly related to the actual time of treatment or age. It is as if the treatment phase itself enforced a new quality of lived time for the patients and unified any pre-existing differences between them—a phenomenon previously described in relation to biographical disruption, altered meaning of time in cancer, and time perspectives^9,12,38^.

The phenomenal explicitness of time around chemo sessions is apparently related to the stress they induce. In this respect, the study confirms and extends findings about time distortions under stress^39^. After chemo, patients' investments of attentional resources appear to be limited, so they prefer watching tv or daydreaming over shopping or social gatherings. There seems to be a preference for emotionally and intellectually less engaging activities. These activities are significantly more frequent than before chemo because patients likely need to wind down from stressful event. Slightly increased engagement in religious practices before chemo may also have grounds in stress as such practices are correlated with coping^40^. Overall, physically more demanding activities (sex, dancing, renovations, sports) are generally much less frequent than other everyday activities (chatting, walking, reading). Reduced interest in such activities before and after chemo sessions may also be caused by similar factors that reduce engagement in activities requiring more attentional resources and emotional arousal.

Theoretically, the sense of time flying and dragging is related to desynchronization between the mind and its environment. Since embodied harmony with the environment and others contributes to well-being while disrupted rhythms confer weakness and vulnerability, such desynchronization is potentially harmful for the patients^41^. Felt time disturbances may be thus interpreted as a challenge for patients' time management skills. This finding opens new perspectives when it comes to preparing patients for particular moments of the chemo cycle. Using more explicit time management approaches to time assessment^26^ may increase patients' awareness of the role of time and help them better engage in daily tasks and responsibilities^42^.

The crucial practical implication comes from the finding that what affects the sense of time dragging and flying around chemo sessions is the very rhythm of chemotherapy, and not its frequency or the time span of therapy. While the rhythmic nature of therapy has an impact on the felt sense of time, it must not put patients' temporal autonomy at risk. We already know that talking to patients about their felt disruptions of time plays a therapeutic role^25^. Both these issues together have significant clinical consequences. Although patients may relatively safely engage in mundane activities and chemo sessions do not drastically destabilise their self-regulation processes in the temporal domain, engaging them in conversations about possible hindrances of temporal experience may be a beneficial addition to therapy.

After chemo patients are slightly more likely to perform activities that do not require physical engagement, such as watching tv and daydreaming. In contrast, before chemo, they instead do things that engage more physical resources, such as taking care of their appearance or their household, shopping, and partaking in social gatherings. It seems reasonable given the rise of physical strength further away from a chemo session. Still, the activities patients do most overall—watching tv, having conversations, going for walks—require only moderate physical and mental engagement. Their choice is understandable given the effects of chemotherapy on patients' energy resources. It thus seems that the management skills of patients before and after chemo are very similar and allow them to engage in everyday activities.

Particular activities undertaken around chemo show that patients' focus is on maintaining basic temporal autonomy rather than increasing their engagement in daily tasks or performance. This is understandable due to the complex psychological and physical burden of chemotherapy. Lessons from the studies of the correlation between time management and stress, e.g.^43,44^. suggest that time management training may reduce this burden. The lack of physical activities right after chemo may be also likely distressing, all the more so since all physically less engaging activities (particularly watching tv and daydreaming) are less capable of filling up the empty time. In this respect, however, there appear to be objective limitations when it comes to clinical ambitions to seek (via training) significant improvement of skills aimed at engagement in daily physical activities. In this respect, mental activities appear to serve patients' needs better.

The impact of mental actions on the body and their complex relation with the felt flow of time interestingly confirms the phenomenological and enactivist views of lived temporality. Consciousness is not independent of movement and one's physical activities, nor merely receptive of the world. It is both embodied and enactive in the world, and hence its intrinsic temporality is also shaped by one's physical movement and environmental engagement^41,45,46^. The felt experience of slowing down may be reinforced by the fact that reduced physical activity (primarily due to the harmful effects of chemo), does not generate desirable changes in one's surroundings. Such a lack of agency could lead to the need to speed up and generate events that would fill the time. The patients’ sense of 'lost' time might thus stem from reduced I-world dynamics, which is beyond personal control due to illness.

The need for acceleration could be satisfied through effectively performed mental activities that are not very energy-consuming but imply a sense of agency (unlike watching tv or daydreaming). On the other hand, the feeling of time flying and not having enough time before chemotherapy could be related to the need to make up for time lost. Patients likely feel improvement in their physical condition and are thus more active. Paradoxically, however, increased physical activity may contribute to the sense of time flying because filling the environment with events' thickens and accelerates it^47^. Replacing some physical activities with mental tasks towards the end of a treatment cycle might thus reduce the feeling of time flying.

Finally, empowering patients to more successfully manage their activities during chemotherapy through careful planning and exercising control over the type of their activities should increase their sense of freedom and autonomy despite the disease. Control is the sense of predictability based on the effects of one's actions in time^48^. Performing even minute actions capable of changing something in a controllable way should give the patients a sense of having free exercisable will beyond the disease and thus empower them in coping with its dreary temporal effects.

### Limitation of the study

Despite the frequencies of chemotherapy being treated as an exposure factor, this study is cross-sectional as far as linear time is concerned. Patients were approached at a specific point in time only once and at distinct stages of their chemotherapy. Furthermore, the survey data was collected during the Covid-19 pandemic, and the patients' overall well-being was likely worse than normal^49^. Also, due to a lack of access to the medical records, it was not possible to determine the stage of cancer (patients themselves were often unable to provide this information) and whether patients were receiving chemotherapy as an adjuvant, neoadjuvant, or sole treatment. The study also lacks data on the adverse effects (e.g., fatigue, nausea) of chemotherapy the patients were experiencing and the medications mitigating those symptoms provided. For this reason, other rhythmic aspects of chemotherapy, in particular the waxing and waning of symptoms and side effects, were not considered, even though they could correlate with the patient's felt sense of time. For instance, chemotherapy-induced nausea and vomiting (CINV) affects up to 70% of the patients and is reported to be the main reason of the quality of life deterioration^50^. Finally, although respondents were out-patient and functional, the sample likely contained some palliative care service users, whose temporal experience, if assessed independently, could have appeared different due to their different therapeutic outlook^51^. Any future studies of felt time during cancer treatment should aim at including these missing medical data.

## Conclusions

The explicitness of time, the felt disruptions of its pace, and losing control over time are common and linked with the very rhythm of chemotherapy regardless of its frequency, duration of treatment, or cancer type. Time flying appears more problematic than time dragging. These feelings do not significantly threaten the temporal autonomy of patients. Still, they are unpleasant, stressful, and not so easy to control. It is, therefore, vital to discuss with patients in chemotherapy the possible disturbances of temporal experience to reduce their adverse effects. The phenomenon of the felt disruptions of time is complex and opens new avenues of clinical research and therapy.

## Data Availability

Data used for this study may be shared upon reasonable request to the corresponding author.
